# Integration of Adverse Outcome Pathways, Causal Networks and ‘Omics to Support Chemical Hazard Assessment

**DOI:** 10.3389/ftox.2022.786057

**Published:** 2022-03-24

**Authors:** Edward J. Perkins, E. Alice Woolard, Natàlia Garcia-Reyero

**Affiliations:** ^1^ Environmental Laboratory, US Army Engineering Research and Development Center, Vicksburg, MS, United States; ^2^ UNC School of Medicine, University of North Carolina Chapel Hill, Chapel Hill, NC, United States

**Keywords:** transcriptomics, networks, toxicogenomics, toxicology, hazard assesment

## Abstract

Several approaches have been used in an attempt to simplify and codify the events that lead to adverse effects of chemicals including systems biology, ‘omics, *in vitro* assays and frameworks such as the Adverse Outcome Pathway (AOP). However, these approaches are generally not integrated despite their complementary nature. Here we propose to integrate toxicogenomics data, systems biology information and AOPs using causal biological networks to define Key Events in AOPs. We demonstrate this by developing a causal subnetwork of 28 nodes that represents the Key Event of regenerative proliferation – a critical event in AOPs for liver cancer. We then assessed the effects of three chemicals known to cause liver injury and cell proliferation (carbon tetrachloride, aflatoxin B_1_, thioacetamide) and two with no known cell proliferation effects (diazepam, simvastatin) on the subnetwork using rat liver gene expression data from the toxicogenomic database Open TG-GATEs. Cyclin D1 (Ccnd1), a gene both causally linked to and sufficient to infer regenerative proliferation activity, was overexpressed after exposures to carbon tetrachloride, aflatoxin B_1_ and thioacetamide, but not in exposures to diazepam and simvastatin. These results were consistent with known effects on rat livers and liver pathology of exposed rats. Using these approaches, we demonstrate that transcriptomics, AOPs and systems biology can be applied to examine the presence and progression of AOPs in order to better understand the hazards of chemical exposure.

## Introduction

Understanding and predicting the potential hazardous effects of chemicals is a major goal in current efforts to protect the health of human and ecological receptors. As a result, numerous strategies have been and continue to be developed to measure and assess chemical hazards. Recent efforts have focused on moving away from traditional animal testing towards the use of *in vitro* assays, biological pathways, and mechanisms of action with adverse outcomes to assess chemical hazards (National Research Council of the National Academies, 2007; Cote et al., 2016). The development and application of inexpensive high throughput and high content assays providing broad coverage of biological effects has also been identified as a critical need in assessing the potential impacts of chemicals (National Academies 2017). The Adverse Outcome Pathway (AOP) framework is one approach that is gaining acceptance in linking biological pathways, represented by *in vitro* assays and other nontraditional toxicological data, to adverse outcomes (Ankley et al., 2010). The AOP framework provides a useful construct in which to document biological pathways and develop predictive models for chemical hazards (Perkins et al., 2019).

The AOP framework is attractive as it creates linear biological pathways composed of essential components, or Key Events (KE), leading to an Adverse Outcome. As a result, AOPs are simplifications of complex biological pathways where KE are measurable biological events. Conversely, systems biology uses detailed descriptions of biological networks (Edwards and Preston 2008; Cote et al., 2016). Systems biology provides the ideal context in which to interpret omics technologies, but its complexity is problematic in a decision-making context (van Ravenzwaay et al., 2017). While the AOP framework and systems biology are complementary, they are generally not combined, limiting the application of ‘omics in a decision-making context.

Since KE are often the result of interactions of several genes, proteins, and/or metabolites, KE provide an opportunity to map the biological networks of systems biology to AOPs in a relatively discrete manner using causal biological networks. AOPs themselves are considered to be linear causal networks where the occurrence of one KE causes the next downstream KE to occur and ultimately cause an Adverse Outcome to occur e.g. a Molecular Initiating Event causes KE1 which causes KE2 which causes an Adverse Outcome A causal subnetwork can also be developed that represents the cascading biological events or nodes required to cause a KE. In other words, the subnetwork represents a network that is contained underneath, or within, a KE. Using a KE causal subnetwork with causal network theory allows one to use ‘omics data to infer the biological state or activity of the overall KE network.

Causal networks can be used to understand the state of individual KE using ideas of conditional dependence/independence from causal network theory (Burgoon, et al., 2017). In causal network theory, specifically the Markov property, the state of a node is only dependent upon the node(s) immediately preceding it, e.g. a predecessor or parent with the causal effect either positive or negative. Building on that is the idea of d-separation, which states that a node is conditionally independent of another node if it is blocked by a third node. As a result, the adverse outcome above would be conditionally independent of the state of KE1 since it is blocked by KE2. Therefore, the adverse outcome will not occur if only KE1 occurs, KE2 must also occur. Using this relationship, if KE2 occurs, then the adverse outcome will occur and can be sufficient to infer the occurrence of an outcome in AOPs. If this can be applied to causal networks representing the molecular events essential for a KE to occur, then this concept may be useful in applying omics to assess if individual KE are occurring in toxicological studies. When combined with dose response analysis, as proposed by Burgoon et al. (2017) causal networks may be useful in a regulatory context to understand at what dose a KE might occur. When applied to multiple KE, this approach might be useful in assessing entire AOPs.

Here, we explore how transcriptomics and systems biology can be merged with AOPs to understand chemicals effect liver biology through the use of causal subnetworks that depict KE in AOPs. We examined the hypothesis that if chemicals are known to cause an adverse effect, regenerative proliferation, and that adverse effect is caused by upstream events (gene/protein activity), then activation of upstream events adjacent to the adverse effect should occur when animals are exposed to chemicals that cause the adverse effect. To do this, we first developed a causal subnetwork for the KE for regenerative proliferation in human/rodent liver using literature-based experimental evidence. We then examined the activation of nodes (genes) in the network in relation to exposure to chemical known to cause regenerative proliferation in rats using five case study chemicals including three carcinogens (carbon tetrachloride, aflatoxin B_1_, and thioacetamide) and two non-carcinogens (diazepam and simvastatin) and data from the toxicogenomics database Open TG-GATEs (Igarashi et al., 2015; https://toxico.nibiohn.go.jp/english/). Our results suggest that the combination of ‘omics and causal subnetworks could be a useful approach to examine the activation of individual KE, and eventually collections of KEs, to better understand the hazards of chemical exposure using the AOP framework.

## Materials and Methods

### Construction of a Causal KE Subnetwork

**FIGURE 1:**
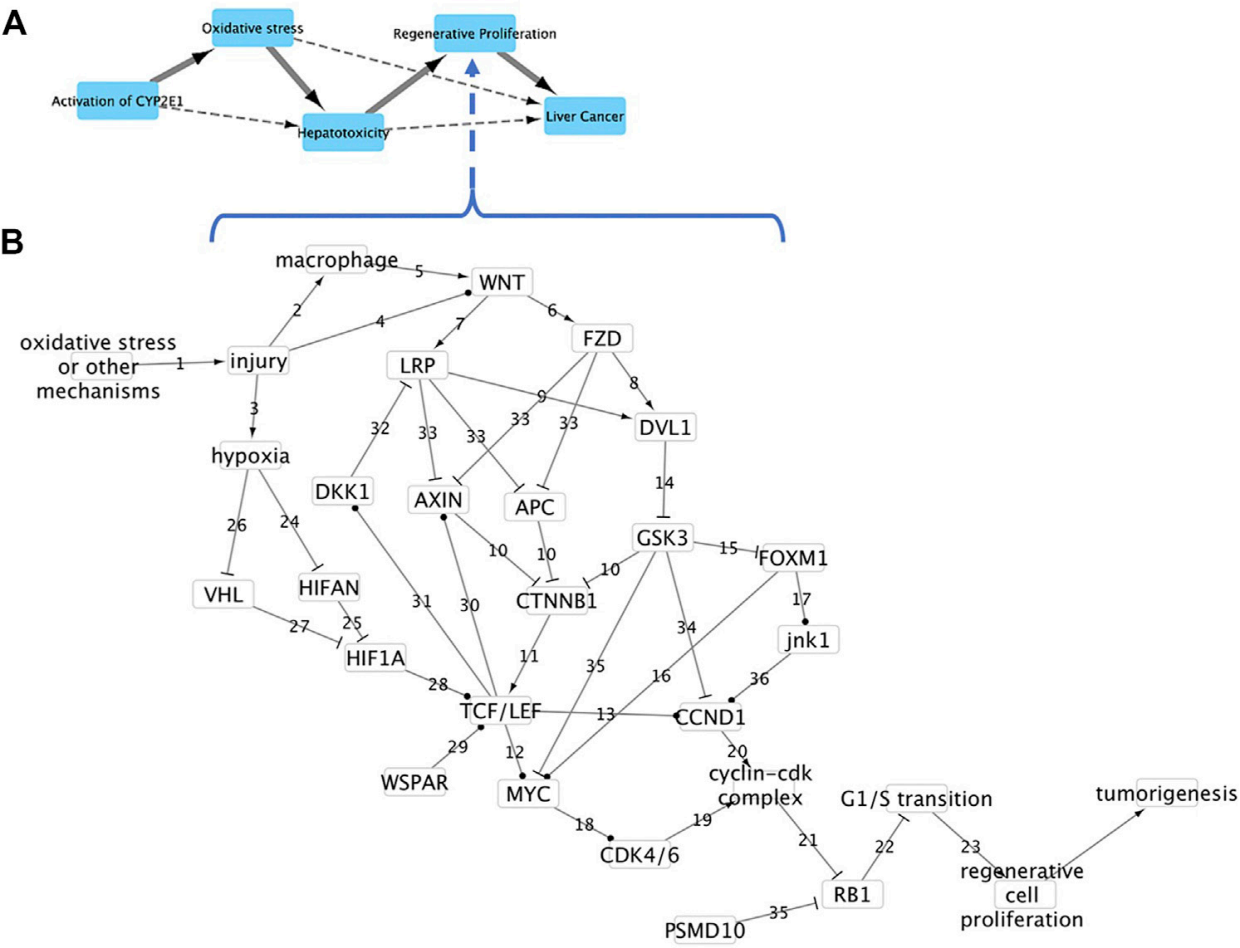
AOP and Key Event causal network leading to liver cancer. **(A)** AOP 220 Cyp2E1 activation leading to liver cancer. Solid arrows represent direct causal relationships between Key Events (KE). Dashed arrows represent indirect relationships. **(B)** Causal subnetwork for KE regenerative proliferation in liver. Nodes represent either genes, proteins or processes. Arrows represent protein activation, “T” sticks represent protein inhibition, ball and sticks represent transcriptional upregulation, and numbers reference action described in main text.

We developed a causal subnetwork for the Key Event of Regenerative Proliferation (Figure 1) that describes how repeated injury to liver tissue can activate Wnt (Wingless-Type MMTV Integration Site Family) and/or hypoxia signaling pathways to dysregulate the cell cycle and lead to regenerative proliferation in the liver. The network was constructed using subject matter expert knowledge driven analysis based on all data available in the peer-reviewed literature and existing pathways available for humans and rodents in the KEGG (https://www.genome.jp/kegg/) and Reactome (https://reactome.org/) databases. Only interactions that where experimental evidence demonstrated causal relationships were used to ensure essentiality in the network. Our confidence that a particular relationship was essential was based on the presence of experimental evidence that the upstream event caused the downstream event to happen (e.g. where down regulation, deletion or silencing of the upstream event blocked the downstream event from happening or when over expression of a gene caused the downstream event to happen). To simplify the network, dose-response and time-dependence factors influencing interactions were assumed to be incorporated into causal linkages, that is, causal linkages incorporate sufficient stimulus and time to needed to develop a full response/activity from the nodes they effect (genes/proteins). The network is freely accessible as the regenerative_proliferation network in the AOP app available in Cytoscape 3 (https://cytoscape.org/; http://apps.cytoscape.org/apps/aopxplorer).

### Gene Expression Analysis

We examined the impact of three chemicals known to induce liver cancer (carbon tetrachloride, aflatoxin B_1_ and thioacetamide) and two non-carcinogenic compounds (diazepam and simvastatin). The effects of these chemicals on livers of rats were assessed using datasets available at Open TG-GATEs (Igarashi et al., 2015; https://toxico.nibiohn.go.jp/). For carbon tetrachloride, gene expression data were analyzed from livers of five rats exposed to a single dose of either 30, 100 or 300 mg/kg carbon tetrachloride and sacrificed 3, 6, 9 or 24 h after exposure. For aflatoxin B_1,_ gene expression data were analyzed from livers of rats exposed to a single dose of either 1, 3 or 10 mg/kg aflatoxin B_1_ and sacrificed after 24 h of exposure. For thioacetamide, gene expression data were analyzed from livers of rats dosed daily with 4.5, 15 or 45 mg/kg thioacetamide until sacrificed at 4, 8, 15, or 29 days of exposure. For diazepam, gene expression data were analyzed from livers of rats dosed daily with either 25, 75 or 250 mg/kg diazepam until sacrificed at 4, 8, 15, or 29 days of exposure. For simvastatin, gene expression data were analyzed from livers of rats dosed daily with either 40, 120 or 400 mg/kg simvastatin until sacrificed at 4, 8, 15, or 29 days of exposure.

All gene expression statistical analyses were performed in R (3.4.3). The median probe intensity from single channel (green channel) microarray data was normalized using robust quantile normalization (PreprocessCore package version 1.38.1). A Bayesian approach was used to identify differentially expressed genes. We focused on expression of 25 genes representing each of the 15 nodes in the regenerative proliferation subnetwork that were detected on the arrays. These were Apc, Axin1, Axin2, Ccnd1, Cdk4, Ctcf, Ctnnb1, Dvl1, Foxm1, Fzd1, Fzd2, Gsk3a, Gsk3b, Lrp5, Lrp6, Myc, Psmd10, Rb1, Vh1, Wnt1, WNT2, Wnt4, Wnt6, Wnt11, Wnt16. We used the rstan (version 2.7.3) package to interface with Stan (http://mc-stan.org/) to perform the Bayesian analyses. For background on the use of Bayesian approaches for gene expression analysis and use of Stan *see* Jiménez-Jiménez et al., 2021.

We used a hierarchical analysis to estimate the treatment effects:

$$\begin{array}{l} y\;∼\;Normal\left(\theta ,\;\sigma \right)\\ \theta =\mu +\tau *\eta \\ \eta ∼\;Normal\left(0,1\right) \end{array}$$

Where y is the estimated treatment effects, σ is the estimated error, and θ is our attempted estimate. We treat θ as a transformed parameter with a default uninformative prior for τ and an uninformative prior of *Normal*(0,1) for η. By using the hierarchical analysis, we allow for some exchangeability of information between the exposures, but not necessarily complete exchangeability. This allows us to use information from all of exposures to inform each other. Note that we are not performing a hierarchical analysis across probes. We used the posterior estimates of θ to identify differentially expressed probes. Specifically, Stan performs a Markov Chain Monte Carlo to estimate the posterior distribution for θ for each site and control. We then calculated the difference of each posterior distribution to obtain the difference distribution. We applied the decision rule stating that differences spanning from log2(1/1.5) to log2(1.5) were functionally equivalent to no change – this is equivalent to using a 1.5-fold-change cut-off – and labeled any region meeting these criteria as a region of practical equivalence (ROPE). If the highest or lowest 95% density interval for the difference distribution is outside of the ROPE, then the probe is differentially upregulated or downregulated, respectively. Else, the probe is not differentially expressed. E.A. Woolard, alice@unc.edu, will provide protocols on this approach upon request.

### Tissue Level Effects of Chemicals

The pathology of rat livers at all exposure times as determined in Open TG-GATEs was used to assess tissue level effects of carbon tetrachloride, aflatoxin B_1_, thioacetamide, diazepam and simvastatin and is available in the Supplemental Tables. In addition to single dose carbon tetrachloride exposures used for gene expression analysis, the pathology of repeated dose exposures to carbon tetrachloride was also examined for effects due to 29 days of daily dosing. For aflatoxin B_1,_ data was only available for the 24 h post exposure treatment groups.

## Results

### Development of a Key Event Causal Subnetwork for Regenerative Proliferation

Here, we describe a KE causal subnetwork of genes, proteins, and processes that, when activated, can lead to regenerative proliferation in humans and rodents. In this network, nodes represent genes, proteins, or processes that are essential for activating regenerative proliferation. In this simplified model, nodes are either on/active or off/inactive and linkages reflect experimentally determined causal relationships that incorporate dose-response and time response relationships between a node/event and its immediate downstream neighbor. All relationships between events have been previously proven to occur and will cause the downstream event to occur given sufficient stimulation and/or time.

Event relationships in the network are numbered in Figure 1 and described below. The network begins with oxidative stress or other mechanisms causing liver tissue injury (event component action or relationship 1 in Figure 1) which in turn causes (2) activation of macrophages and wound repair (Boulter et al., 2012), (3) increased hypoxia through diminished blood supply or activity of reactive oxygen species (Ju et al., 2016; Gonzalez et al., 2018) and (4) increased expression of Wnt ligands (Okabe et al., 2016). The activation of macrophages causes (5) activation of Wnt proteins and Wnt signaling (Boulter et al., 2012; Vannella and Wynn 2017). The activation and/or increased expression of Wnt signaling ligands causes (6) binding of the Wnt ligand to the co-receptors Frizzled (Fzd family) and (7) Low-density lipoprotein receptor-related proteins 5 and 6 (Lrp5/6) which then (8) recruit and phosphorylate Dishevelled (Dvl1) and the scaffold protein Axin (Takigawa and Brown 2008).

The phosphorylation and recruitment of Axin (Axin1, Axin2) (33) inhibits formation of the beta-catenin destruction complex, composed of Axin1 or Axin2, adenomatosis polyposis coli (APC), beta-catenin (Ctnnb1) and glycogen synthase kinase 3 (Gsk3), which (10) targets beta-catenin for degradation. Inhibiting formation of the destruction complex increases the amount of available beta-catenin to (11) interact and complex with the transcription factor 7 and lymphoid enhancer-binding factor (Tcf/Lef) family of transcription factors (Tf7, Tcf7l1, Tcf7l2, Lef1; Takigawa and Brown 2008). The Tcf/Lef:beta-catenin complex then (12) activates transcription of Myc proto-oncogene (Myc) and (13) cyclin D1 or Ccnd1 (Schuijers et al., 2014; Katoh 2017). Activation of Wnt signaling (14) inhibits Gsk3 phosphorylation activity which then (15) represses forkhead box M1 (Foxm1) activity, (34) causes increased turnover of Ccnd1 and (35) increases proteolysis of Myc (Katoh 2017). Activation of Wnt signaling (14) inhibits Gsk3 phosphorylation activity which (15) represses forkhead box M1 (Foxm1), (34) causes increased turnover of Ccnd1 and (35) increased proteolysis of Myc (Gregory et al., 2003).

Foxm1 activates (16) transcription of Myc and (17) transcription of Mapk8, the mitogen-activated protein kinase (also known as Jnk1; Wierstra and Alves 2007; Wang et al., 2008). Transcriptional activation of Mapk8 then leads to (36) transcriptional activation of Ccnd1 (Wang et al., 2008). Transcriptional activation of Myc causes (18) transcription of cyclin-dependent kinase 4 and 6 (Cdk4/6) which leads to (19, 20) formation of a Cdk4/6 and Ccnd1 complex (Wang et al., 2011). The cyclin-Cdk complex then (21) inhibits activity of the retinoblastoma (Rb1) transcriptional corepressor 1 which (22) negatively regulates the cell cycle (Burkhart and Sage 2008). Dysregulation of G1/S transition by inhibition of Rb1 and/or Foxm1 (23) leads to cell proliferation (Wierstra and Alves 2007; Burkhart and Sage 2008).

Myc can also be activated *via* hypoxia signaling where an increase in hypoxia (24) decreases the activity of oxygen sensor hypoxia-inducible factor 1 alpha inhibitor (Hif1an) thereby reducing the ability of Hif1an to (25) hydroxylate and inhibit hypoxia-inducible factor 1 alpha (Hif1a) activity (Mahon et al., 2001; Whyte et al., 2012). Hypoxia also can (26) inhibit activity of the von Hippel-Lindau (Vhl) tumor suppressor protein which has been shown to (27) hydroxylate Hif1a in an O_2_ dependent manner marking Hif1a for degradation and inactivation in addition to inhibiting expression of Hif1A (Mahon et al., 2001). In stem cells, activated Hif1a (28) increases expression of Tcf/Lef leading to increased expression of genes including Myc (Whyte et al., 2012; Tiburcio et al., 2014).

The long noncoding RNA WSPAR is often highly expressed in human hepatocellular carcinoma cells and has been found to (29) activate expression of members of the Tcf/Lef family (Zhan et al., 2017). Tcf/Lef transcription factors (30) increase transcription of Axin2 and increase destruction of beta-catenin in a Wnt signaling negative feedback loop (Jho et al., 2002). Tcf/Lef transcription factors form a negative feedback loop that inhibits Wnt signaling by (31) activating transcription of the dickkopf Wnt signaling pathway inhibitor 1 (Dkk1) which then (32) binds to the LRP co-receptor (Takigawa and Brown 2008). Finally, cellular G1/S transition can also be dysregulated by (35) phosphorylation of Rb1 by the 26S proteasome non-ATPase regulatory subunit 10 (Psmd10) which results in an increase in proteosomal degradation of Rb1 (Higashitsuji et al., 2005).

#### Subnetwork Events Sufficient to Infer Regenerative Proliferation

Identification of genes that can be used to infer that regenerative proliferation is occurring would be valuable in determining if the KE were activated and could cause hepatocellular carcinoma given sufficient time and stimulus. Here we used the approach of Burgoon et al. (2017) to identify a small set of genes that are sufficient to identify that the KE is likely to occur. Sufficiency was defined by Rothman (1976) as a “cause which inevitably produces the effect.” In Rothmans’ sufficient-component cause model, there are one or more of the network components that are sufficient, either individually or jointly, to infer an outcome/disease. Removal of one of these components is sufficient to stop progression to an adverse outcome. In the causal subnetwork, if a downstream event (e.g. regenerative proliferation) is affected by a chemical, then the events upstream must have happened. As a result, if a sufficient event has been identified and the occurrence of this event is sufficient to infer an outcome, (regenerative proliferation), then if event occurs, we can infer that the outcome will occur.

Sufficient events were identified using the Markov property of Causal network theory, which states that a node is only dependent upon its predecessor or parent and using the idea of d-separation, which states that a node is conditionally independent of another node if it is blocked by a third node. For example, in our network, Rb1 is dependent upon Cdk4/6 and Ccnd1 but is conditionally independent of Myc (Figure 1). Using these criteria, we identified Ccnd1, Rb1, Cdk4/6 and Psmd10 as sufficient events, measurable with gene expression, that can infer dysregulation of G1/S transition and ultimately regenerative proliferation in livers of exposed animals (Figure 1).

This is validated by experimental and pathological evidence demonstrating that if any of these four genes/proteins are dysregulated, cell proliferation, and eventually cancer, will occur given continued dysregulation. Ccnd1 gene expression is well documented to control cell cycle progression and control cancer development in humans and rodents (Marampon et al., 2016). Moreover, silencing of Ccnd1 expression in human cell lines suppresses cell proliferation (Ding et al., 2020). Loss of Rb1 has been found to cause cell proliferation and tumor formation in humans, while expression of Rb1 eliminates cell proliferation caused by loss of Rb1 in humans cells and mouse models (Chicas et al., 2010; Zhu et al., 2015; Doan et al., 2021). Inhibition of CDK4/6 has been shown to arrest cell-cycle progression in human hepatoma cells and mice (Rivadeneira et al., 2010). PSMD10 levels are elevated during human hepatocarcinogenesis and silencing of Psmd10 expression repressed cell proliferation and tumorigenicity in human HepG2 cells (Jing et al., 2014).

### Case Studies of Effects of Chemicals Known to Cause Regenerative Proliferation on Subnetwork in Rat Livers

The regenerative proliferation subnetwork represents essential biological events that occur as liver cellular injury causes cellular proliferation. As such, the network reflects the biology occurring at the time of analysis and does not identify whether or not a chemical is carcinogenic. To examine how the biological subnetwork behaved as a result of liver damage, we examined the effect of three chemicals well documented to cause regenerative proliferation and two chemicals that do not cause regenerative proliferation. When protein interactions were depicted in the network, we used an increase in gene expression as an approximate surrogate for protein activity in the absence of other information with the recognition that increased gene expression does not necessarily reflect protein activity temporally or in abundance. Activated/inhibited genes/proteins were assumed to cause downstream effects given sufficient time/stimulation.

#### Carbon Tetrachloride

Carbon tetrachloride is a well-studied model toxicant known to cause liver damage, resulting in fatty degeneration, cellular necrosis, fibrosis, regenerative proliferation and, given sufficient time, cancer (Manibusan et al., 2007). Liver pathology data was examined for evidence of liver injury, necrosis, steatosis, cell proliferation and tumor formation (Supplementary Table S1). At 24 h, pathology data indicates that the 300 mg/kg carbon tetrachloride dose group had significant evidence of inflammation (cellular infiltration), cellular injury (hypertrophy) and steatosis (fatty degeneration); the 100 mg/kg group had a moderate (two of five animals) incidence of cellular injuries and steatosis while the 30 mg/kg treatment group had no effects. Effects on animals exposed to repeated doses of 300 mg/kg for 29 days provides evidence of long-term effects of carbon tetrachloride. At 29 days, livers of all animals displayed inflammation, cellular injury, steatosis and fibrosis.

**FIGURE 2:**
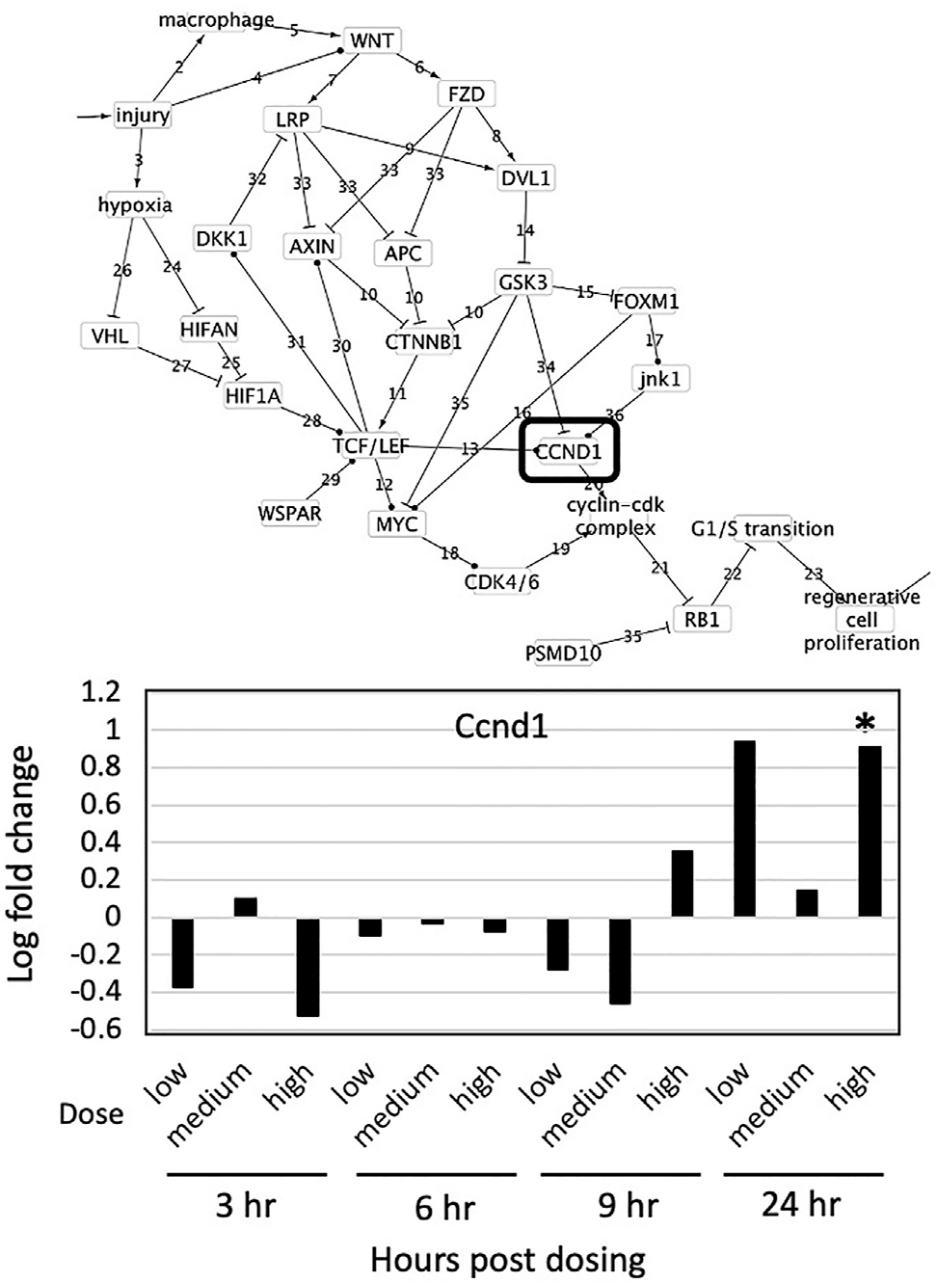
Effect of carbon tetrachloride on expression of genes found in the causal subnetwork for regenerative proliferation in rat liver. Gene expression data was analyzed from livers of rats exposed to a single dose of either low (30 mg/kg), medium (100 mg/kg) or high (300 mg/kg) doses of carbon tetrachloride and sacrificed after 3, 6, 9 or 24 h of exposure. Gene expression values significantly different from controls are denoted with asterisks in the chart and by a box in the subnetwork diagram.

Of all genes present in the regenerative proliferation subnetwork and across all exposures, only expression of Ccnd1 was upregulated and only at 300 mg/kg with 24 h exposure (Figure 2). In mice exposed to carbon tetrachloride, McCracken et al. (2017) also found that Cnnd1 gene expression was upregulated in livers before pathological evidence of cell proliferation at the organ level changes was detectable. Based on the sufficiency of Ccnd1, we can infer that cellular proliferation is occurring consistent with the known toxicity of carbon tetrachloride and the progression of tissue level effects seen in exposed animals.

CCND1 is an essential protein that forms a complex with Cdk4/6 which regulates cell cycle G1/S transition (Figure 1, event component action 19–22). Ccnd1 is generally transcriptionally regulated, with upregulation resulting in cell proliferation (O’Leary et al., 2016). In the causal subnetwork, regenerative proliferation is conditionally dependent upon CCND1, and Ccnd1 d-separates regenerative proliferation from all other nodes with the exception of the Cyclin-Cdk complex and Rb1 (Figure 1). We would not expect to see changes in Rb1 gene expression as the protein, pRb, is generally post-transcriptionally regulated, and cyclin D1 is the protein that regulates/signals the destruction of pRb (Alao 2007). Therefore, measurement of increased expression of Ccnd1 is sufficient to infer dysregulation of G1/S transition and ultimately that regenerative proliferation is likely to occur.

#### Aflatoxin B_1_

Aflatoxin B_1_ is the most potent hepatotoxic and hepatocarcinogenic molecule of the aflatoxins, produced naturally by *Aspergillus* molds (Rushing and Selim 2019). The liver pathology of animals 24 h after exposure to a single dose of 1, 3 or 10 mg/kg aflatoxin B_1_ was examined for evidence of liver injury, necrosis, steatosis, cell proliferation and tumor formation (Supplementary Table S2). Animals exposed to 1 mg/kg aflatoxin B_1_ displayed slight cell necrosis. At 3 mg/kg aflatoxin B_1_ animals displayed a slight to moderate degree of inflammation and necrosis. 24 h after 10 mg/kg aflatoxin B_1_ exposure, animals displayed effects of inflammation, severe cell necrosis and a slight degree of atrophy.

**FIGURE 3:**
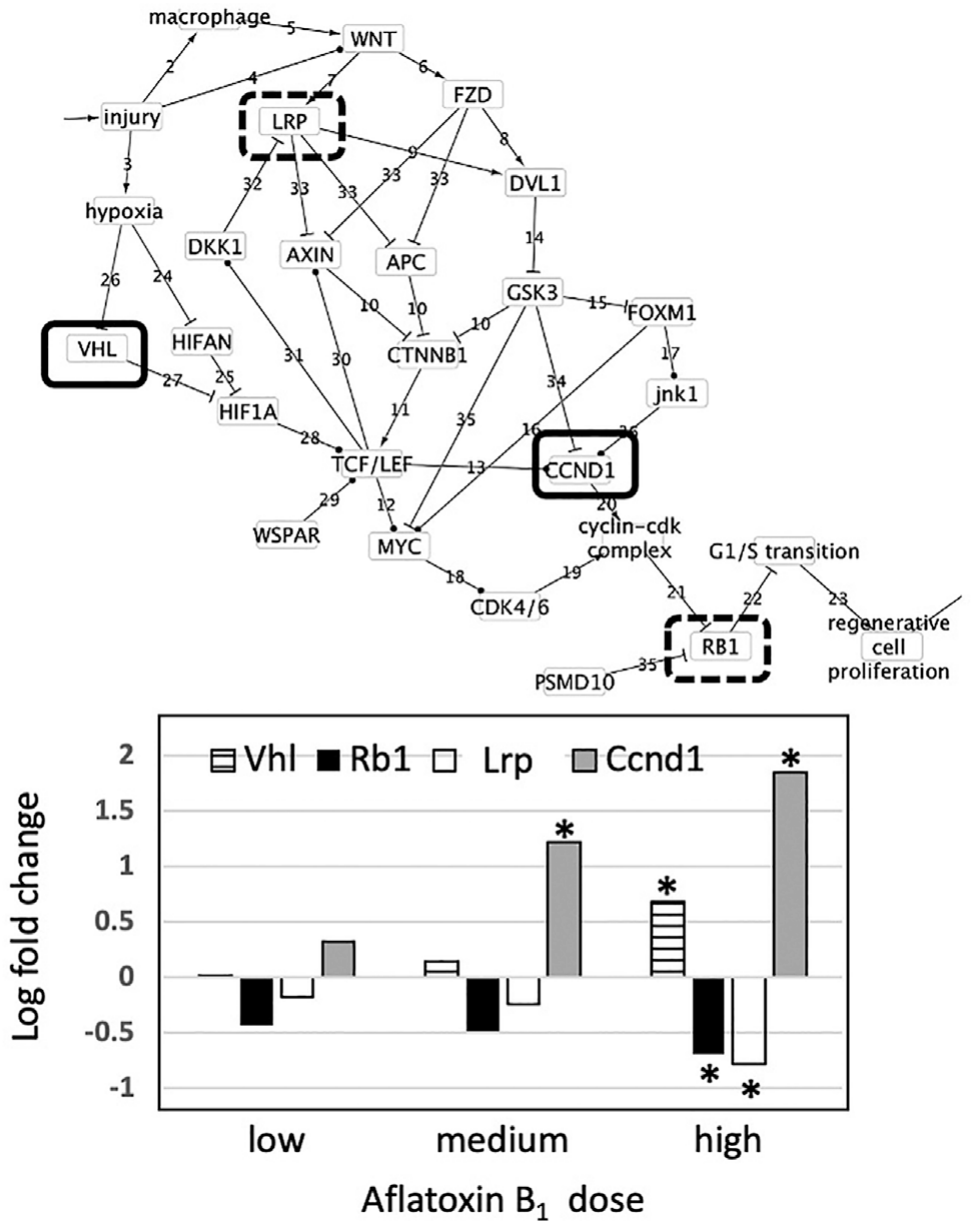
Effect of aflatoxin B_1_ on expression of genes found in the causal subnetwork for regenerative proliferation in rat liver. We analyzed gene expression data from livers of rats exposed to a single low (1 mg/kg), medium (3 mg/kg) or high (10 mg/kg) dose of aflatoxin B_1_ that were sacrificed after 24 h of exposure. Expression of the gene Vhl is denoted by a hatched column, Rb1 by a black column, and Ccnd1 by a grey column in the chart. Gene expression values significantly different from controls are denoted with asterisks in the chart. Upregulated genes are denoted by a solid lined box in the subnetwork diagram and downregulated genes by dashed box.

Gene expression results were consistent with pathology results. Across all genes and all exposures, only Ccnd1, Rb1, Lrp, and Vhl were differentially expressed. Expression of genes increased/decreased with increasing dose of Aflatoxin B_1_ and severity of tissue level response. Ccnd1 was upregulated in both the medium 3 mg/kg and high 10 mg/kg treatment groups. Both Lrp5 and Rb1 were downregulated while Vhl was upregulated in the 10 mg/kg treatment group (Figure 3). Since Ccnd1 and Rb1 are sufficient to infer the outcome, we can infer cell proliferation is occurring. This is consistent with pathology findings indicating that cell injury and death at the tissue level is occurring, as these precede manifestation of cell proliferation at the tissue level.

As described previously, CCND1 works with pRb to play an essential role in regenerative proliferation. While not always expected due to pRb being post-transcriptionally regulated, we still see downregulation in the Rb1 gene at the highest aflatoxin B_1_ dose. The changes observed in these two genes are consistent with the regenerative proliferation network we have developed. Depending on oxygen availability conditions, VHl either interacts with the Myc pathway or initiates angiogenesis (Maxwell and Ratcliffe 2002). However, the high number of intermediary events at the protein-level between VHl and G1/S phase transition dysregulation preclude this gene change from being causal in the regenerative proliferation pathway (Figure 3). Likewise, though its downregulation has been associated with chronic renal failure, Lrp cannot be causal due to its distance from the key endpoint (Kim and Vaziri, 2005). While the dysregulation seen in Lrp and Vhl is not causal in the described pathway, the changes shown in Ccnd1 and Rb1 are sufficient to infer dysregulation of G1/S transition, which yields regenerative cell proliferation.

#### Thioacetamide

Thioacetamide is a synthetic compound used to replicate the progression of liver disease in animal models due to its well-characterized hepatotoxicity (Al-Bader et al., 2000; Ingawale et al., 2014). The liver pathology of animals exposed daily for 4, 8, 15 or 29 days to 45 mg/kg was examined for evidence of liver injury, necrosis, steatosis, cell proliferation, fibrosis and tumor formation (Supplementary Table S3). After 4 days, all animals displayed inflammation, cell injury, and cell necrosis. After 8 days, all animals displayed nuclear alteration, inflammation, cell injury, and minimal to slight degree of cell necrosis. After 15 days, all animals displayed nuclear alteration, cell injury, a minimal degree of oval cell proliferation and two of five animals had a minimal degree of cellular foci. After 29 days, all animals displayed a severe degree of nuclear alterations, a slight to moderate degree of cellular foci, a severe degree of eosinophilic granular degeneration; a minimal degree of fibrosis, a moderate degree of hypertrophy, a moderate degree of bile duct proliferation and a slight degree of oval cell proliferation. These observations indicate the development of liver injury, necrosis, cell proliferation and fibrosis with increasing dose and time of exposure to thioacetamide.

**FIGURE 4:**
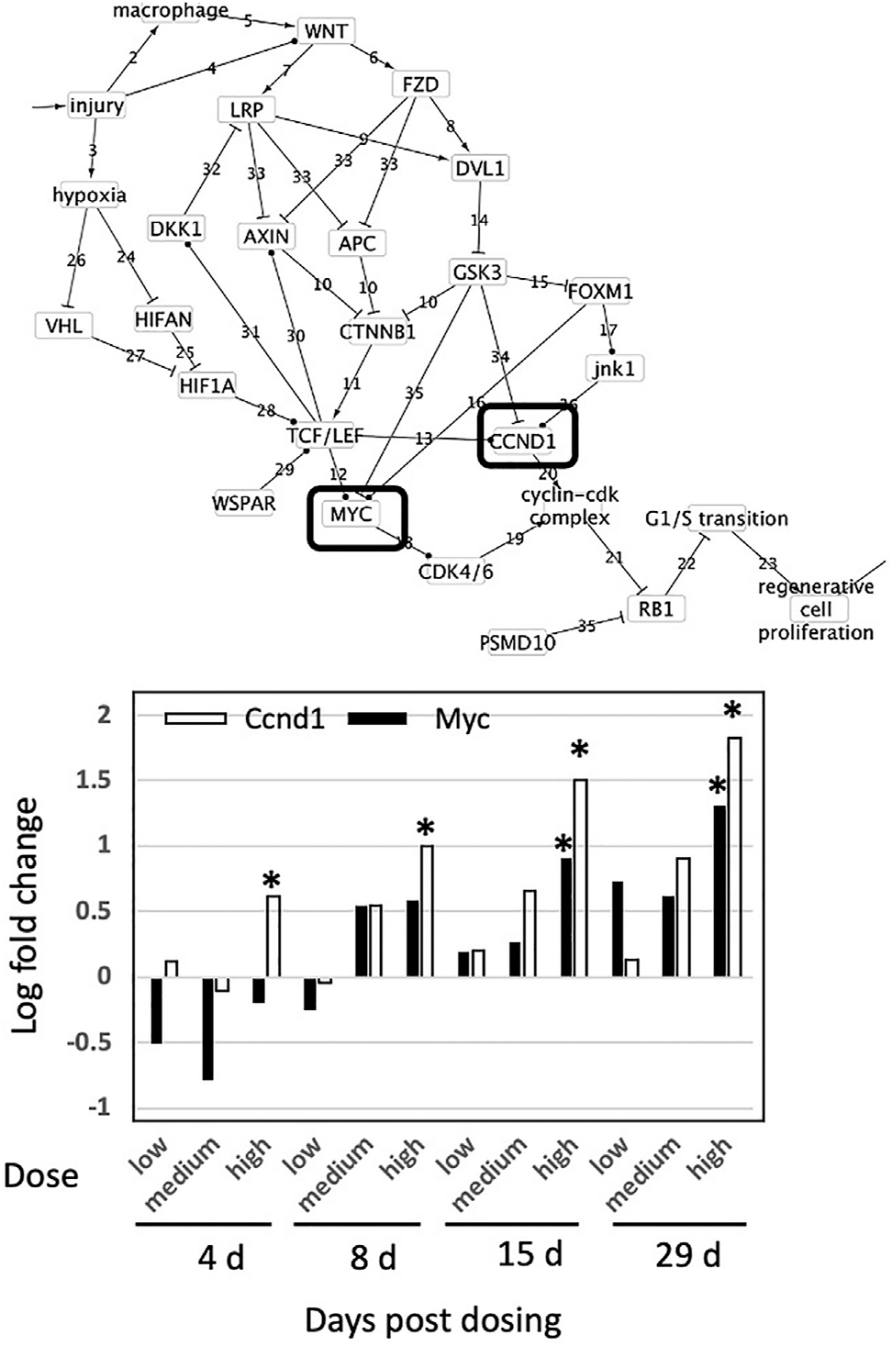
Effect of thioacetamide on expression of genes found in the causal subnetwork for regenerative proliferation in rat liver. We analyzed gene expression data from livers of rats exposed to a single low (4.5 mg/kg), medium (15 mg/kg) or high (45 mg/kg) dose of thioacetamide and sacrificed after 4, 8, 15, or 29 days of exposure. Expression of the gene Ccnd1 is denoted by a white column and Myc by a black column in the chart. Gene expression values significantly different from controls are denoted with asterisks in the chart. Upregulated genes are denoted by a solid lined box in the subnetwork diagram.

Across all genes and all exposures, only Ccnd1 and Myc were affected (Figure 4). Expression of both Ccnd1 and Myc increased with increasing exposure time. Ccnd1 was upregulated in the 45 mg/kg dose at all four durations of exposure. Myc was upregulated in the 45 mg/kg dose at 15 and 29-day durations of exposure. Activation of Ccnd1 indicates that cell proliferation is occurring in the rat liver in response to thioacetamide beginning at day 4, preceding evidence of cell proliferation at the tissue level, oval cell and bile duct proliferation, beginning at day 15 (Fausto and Campbell 2003; Sato et al., 2019).

Because CCND1 forms a complex with CDK4/6 that regulates cell cycle G1/S transition (Wang et al., 2011), regenerative proliferation is conditionally dependent upon CCND1, and CCND1 d-separates regenerative proliferation from all other nodes with the exception of the Cyclin-Cdk complex and Rb1. pRb is generally post-transcriptionally regulated, and cyclin D1 regulates/signals destruction of pRb (Alao 2007). Therefore, measurement of increased expression of Ccnd1 is sufficient to infer dysregulation of G1/S transition and ultimately that regenerative proliferation is likely to occur.

#### Diazepam

**FIGURE 5:**
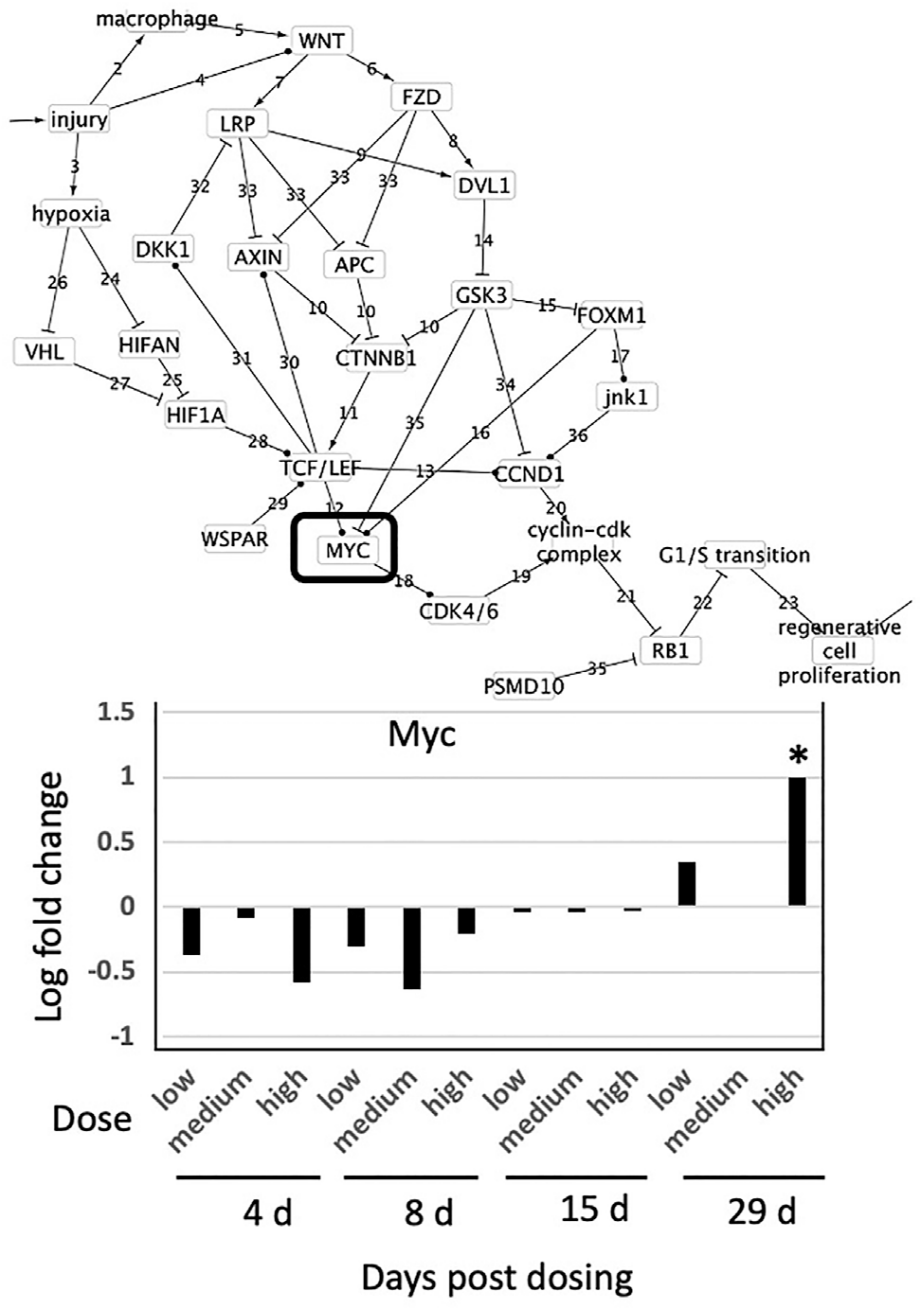
Effect of Diazepam on expression of genes found in the causal subnetwork for regenerative proliferation in rat liver. We analyzed gene expression data from livers of rats exposed to a single low (25 mg/kg), medium (75 mg/kg) or high (250 mg/kg) dose of diazepam and sacrificed after 4, 8, 15, or 29 days of exposure. Gene expression values significantly different from controls are denoted with asterisks in the chart and by a box in the subnetwork diagram.

Diazepam is a benzodiazepine commonly used to treat anxiety, and has no known relation to liver cancer progression (Diazepam 1996). Analysis of liver pathology of animals exposed daily to either 25, 75 or 250 mg/kg diazepam for 4, 8, 15 and 29 days indicated no effects at 25 mg/kg diazepam and that 75 mg/kg caused no effects other than cell injury in two of five animals after 29 days exposure (Supplementary Table S4). For the 250 mg/kg treatment group, all lengths of exposure displayed evidence of increased cell size (hypertrophy) with the exception of one animal that displayed a minimal degree of fatty degeneration at 4 days exposure. Across all genes and all exposures, only Myc was differentially expressed and only at 250 mg/kg and 29-day exposure (Figure 5). Gene expression indicates that cell proliferation is unlikely to be occurring, as no genes sufficient to infer cell proliferation were affected and cell proliferation is conditionally independent from Myc activity. This is consistent with the liver pathology and known effects of diazepam on the liver.

#### Simvastatin

Simvastatin is an HMG CoA reductase inhibitor commonly prescribed to patients to treat high cholesterol and triglyceride levels (Duarte et al., 2021). This compound has no documented direct role in liver carcinogenesis. Analysis of liver pathology of animals exposed daily to the highest dose of 400 mg/kg for 4, 8, 15 or 29 days (Supplementary Table S5) identified no pathological features of liver steatosis, regenerative proliferation, or hepatocellular carcinomas in animals exposed to simvastatin. Histopathological features indicating injury (increased mitosis) or inflammation (basophilic change, microgranuloma) were observed. Across all genes and all exposures, no gene was differentially expressed, and therefore there was no evidence for activation of the regenerative proliferation pathway. This is consistent with the liver pathology and known effects of simvastatin on the liver.

## Conclusion

Here we propose the development of a simple systems biology, causal subnetwork to represent critical events within a KE in order to define and understand the state of a KE using omics and systems biology approaches. It should be noted that we do not use the causal network to predict if a chemical causes liver injury/regenerative cell generation. We use the network to measure the state of the tissue after exposure. By developing the causal network, we were able to identify essential events leading from tissue damage to dysregulation of cell division and genes/proteins whose activity is sufficient to indicate activation of the KE Regenerative proliferation.

We have intentionally focused only on events within an individual KE rather interactions between KE to maintain the hierarchy where an AOP organizes KE and KE organize subevents/subnetworks. To illustrate how such a subnetwork could be developed and applied, we developed a KE subnetwork that describes the development of regenerative cell proliferation due to repeated liver injuries from several mechanisms including oncotic necrosis, chronic inflammation and oxidative stress caused by electrophilic metabolites from cytochrome p450 metabolism of xenobiotics (Smith et al., 2016). The subnetwork represents a common KE in cancer AOPs such as AOP220 *Cyp2E1 activation leading to liver cancer* (https://aopwiki.org/aops/220; Figure 1). The regenerative proliferation subnetwork (Figure 1) is composed of 28 events (genes, proteins or processes). In this simplified network model, events are considered either as on/active or off/inactive, connections are causal (activate/promote, inactivate/inhibit), dose-dependence and time dependence are inherent characteristics of the causal linkage. However, dose-response features could be incorporated into an event using point of departure analysis of gene expression or assay activity as described in Burgoon et al. (2017).

Several assumptions were made in applying the subnetwork. Parent events have been shown to cause daughter event, therefore if a parent event occurs the daughter event will also occur. Since the effect of time and dose dependence is not explicitly accounted for in network interactions but the linkage is causal, activated/inactivated events were assumed to cause their nearest downstream event given sufficient time and stimulus. To demonstrate how omics, or transcriptomics, can be used to monitor activity in the subnetwork, we used gene expression as a surrogate to assess if a protein or gene changed levels/activity, realizing that this does not necessarily reflect protein activity temporally or in abundance and may not capture certain protein interactions represented in the subnetwork.

The subnetwork was used to identify events (genes or proteins) whose activity could be used to infer the occurrence of the outcome (the KE regenerative proliferation) using causal network theory. Five proteins were identified as sufficient to infer regenerative proliferation events based on their proximity to cell proliferation: Cyclin D1 (CCND1), RB transcriptional corepressor 1 (pRb), proteasome 26S subunit, non-ATPase 10 (PSMD10) and Cyclin-dependent kinase 4 and 6 (CDK4/6). These were confirmed in the scientific literature as sufficient to infer cell proliferation as removal of action (activation or inhibition) of these proteins prevents the occurrence of cell proliferation (Rivadeneira et al., 2010; Jing et al., 2014; Zhu et al., 2015; Marampon et al., 2016; Ding et al., 2020; Chicas et al., 2010; Doan et al., 2021). Activity of these genes/proteins were used to infer whether or not regenerative cell proliferation was occurring in liver tissue.

We examined the behavior of the subnetwork in conditions that cause liver injury and promote regenerative cell generation by examining livers of rats exposed to chemicals with well characterized effects on liver injury and regenerative proliferation (carbon tetrachloride, aflatoxin B_1_, thioacetamide) and chemicals with no known effect on liver injury and regenerative proliferation (diazepam and simvastatin). The behavior of genes in the network were consistent with liver pathology data for tissue level effects related to hepatocellular carcinoma development and known effects of the chemicals on liver. Genes sufficient to infer cell proliferation were affected only where animals were exposed to chemicals known to cause liver injury and cell proliferation. Interestingly, gene expression sufficient to infer that cell proliferation was occurring was detectable much earlier than effects on the tissue levels. This is expected given that changes at the gene expression levels frequently occur before changes at the tissue level.

These analyses demonstrate that the combination of transcriptomics and causal subnetworks could be used to describe or infer the sate of a KE and provides a promising approach to merge omics, systems biology and AOPs. Measuring sufficient sets of genes in causal networks for Key Events in AOPs or AOP networks can provide more efficient and informative measurements related the toxicity or Adverse Outcome of a chemical. Applying causal theory reduces the uncertainty associated with measuring an event distal from the adverse outcome and interfering communication from other pathways. If combined with targeted sequencing, transcriptomics-based point of departure and high throughput assays this approach could greatly facilitate our understanding of adverse effects caused by chemicals. Future research into the utility and applicability of this approach should test to see if evidence from *in vitro* liver cell systems (and the causal network presented here) are accurate predictors of damage/injury in whole organs/animals. Additional research is needed to determine if combining the activity of sufficient genes in reference chemical exposures with classification modeling be useful in identifying chemicals that activate this KE, if these causal subnetworks are sensitive and/or specific enough to be used in screening for chemical effects and whether or not the subnetworks serve to organize and explain existing data rather than make prospective predictions of *in vivo* effects using *in vitro* data.

## Supplemental Tables

Table 1. Liver pathology data for carbon tetrachloride exposures. Low = 30 mg/kg, medium = 100 mg/kg and high = 300 mg/kg. Data for individual rats are designated by glass image numbers and highlighted by yellow or white rows.Table 2. Liver pathology data for aflatoxin B_1_ exposures. Low = 1 mg/kg, medium = 3 mg/kg and high = 10 mg/kg. Data for individual rats are designated by glass image numbers and highlighted by yellow or white rows.Table 3. Liver pathology data for thioacetamide exposures. Low = 4.5 mg/kg, medium = 15 mg/kg and high = 45 mg/kg. Data for individual rats are designated by glass image numbers and highlighted by yellow or white rows.Table 4. Liver pathology data for diazapam exposures. Low = 25 mg/kg, medium = 75 mg/kg and high = 250 mg/kg. Data for individual rats are designated by glass image numbers and highlighted by yellow or white rows.Table 5. Liver pathology data for simvastatin exposures. Low = 40 mg/kg, medium = 120 mg/kg and high = 400 mg/kg. Data for individual rats are designated by glass image numbers and highlighted by yellow or white rows.

## Data Availability

Publicly available datasets were analyzed in this study and can be found in Igarashi et al. (2015). The Array data are available at https://www.toxicodb.ca/datasets/2.
